# Exploring the Acceptability of Expanded Perinatal Depression Care Practices Among Women Veterans

**DOI:** 10.1007/s11606-022-07573-7

**Published:** 2022-08-30

**Authors:** Aimee Kroll-Desrosiers, Rebecca L. Kinney, Valerie Marteeny, Kristin M. Mattocks

**Affiliations:** 1 VA Central Western Massachusetts Healthcare System, Leeds, MA USA; 2 University of Massachusetts Chan Medical School, Worcester, MA USA

**Keywords:** perinatal depression, postpartum depression, antenatal depression, Veterans, mental health counseling

## Abstract

**Background:**

Veterans receive obstetrical care from community-based providers contracted through the Veterans Health Administration (VA); however, Veterans remain eligible for VA mental healthcare in the perinatal period. To date, few studies have focused specifically on the mental health needs of Veterans during the perinatal period.

**Objective:**

To examine the acceptability of more comprehensive perinatal mental healthcare screening and treatment in VA care, we explored pregnant and postpartum Veteran perspectives of United States Preventive Services Task Force (USPSTF) recommendations that aim to expand mental health counseling for the prevention and treatment of perinatal depression.

**Design:**

Semi-structured interviews with pregnant and postpartum Veterans enrolled in VA care, integrated with quantitative survey data.

**Participants:**

Pregnant and postpartum Veterans (*n*=27) who had delivered infants or were due by February 2020.

**Approach:**

Framework analysis with an inductive approach was utilized to understand our data, interpret and code our transcripts, and develop themes.

**Key Results:**

Fewer than half (44%) of the women reported seeing a mental health provider at the beginning of their pregnancy. We found that Veterans support USPSTF recommendations in the VA, consider mental healthcare to be very important during the perinatal period, would like better access to mental healthcare resources and peer support networks, and suggest that perinatal depression screening could be more extensive.

**Conclusions:**

These findings support the implementation of more comprehensive perinatal depression prevention policies and practices within VA care. Understanding the real-world feasibility and prevailing barriers to comprehensive perinatal depression care is needed to inform implementation of the USPSTF recommendations or a similar intervention tailored for VA care.

**Supplementary Information:**

The online version contains supplementary material available at 10.1007/s11606-022-07573-7.

## BACKGROUND

Over the past decade, the Veterans Health Administration (VA) has paid for over 52,000 deliveries for Veterans.^1^ Maternity care is provided through contracted community obstetrical providers enrolled in the VA Community Care Network.^2^ Throughout pregnancy, VA maternity care coordinators offer pregnancy resources and support to Veterans,^3^ and Veterans remain eligible for VA mental healthcare. Access to perinatal mental healthcare is important as depression is the most common complication of pregnancy.^4^ Psychotherapy has been shown to improve perinatal depression outcomes,^5–7^ and early engagement with counseling may address some of the existing barriers to perinatal mental healthcare, such as stigma about seeking depression treatment during pregnancy or the postpartum period.^8^

In 2019, the United States Preventive Services Task Force (USPSTF) issued recommendations that “clinicians provide or refer pregnant and postpartum women who are at increased risk of perinatal depression to counseling interventions (e.g., cognitive behavioral therapy, interpersonal therapy) to prevent episodes of depression.”^9,10^ While there is no accurate screening tool to identify perinatal depression risk, the USPSTF suggests providing counseling to women with 1 or more of the following: a history of depression, socioeconomic risk factors such as low income or single parenthood, recent intimate partner violence, or mental health-related factors such as elevated anxiety symptoms or a history of significant negative life events.

Incorporating the USPSTF recommendations into VA practice may be an important step in preventing perinatal depression in Veterans. Among pregnant Veterans, depression symptoms (as measured by an Edinburgh Postnatal Depression Scale (EPDS) score of 10 or greater) were present in nearly 1/3 of a prenatal sample, higher than typical prevalence estimates in civilians.^11^ Furthermore, Veterans are at increased risk of other predictors of perinatal depression, including homelessness,^12^ unemployment,^13^ a history of traumatic life events,^14–16^ and intimate partner violence, which nearly one-third of women Veterans report experiencing.^17–19^ Factors associated with perinatal depression that are specific to military service are also common and include experiences of military sexual trauma, combat-related trauma, and post-deployment stressors.^20^ These factors highlight the need for adequate perinatal mental healthcare, as perinatal depression symptoms in Veterans often remain underrecognized and undertreated.^21,22^

To date, no studies have specifically identified whether Veterans are interested in greater availability of mental healthcare, such as counseling as indicated by the USPSTF recommendations, from the VA in the perinatal period. Therefore, the objective of this study was to explore Veterans’ perspectives on implementing USPSTF recommendations that would refer them to a VA mental health counselor at the time of pregnancy confirmation, reflecting on their recent and ongoing experiences of mental healthcare and depression screening during the perinatal period.

## METHODS

### Sample Selection

**Fig. 1:**
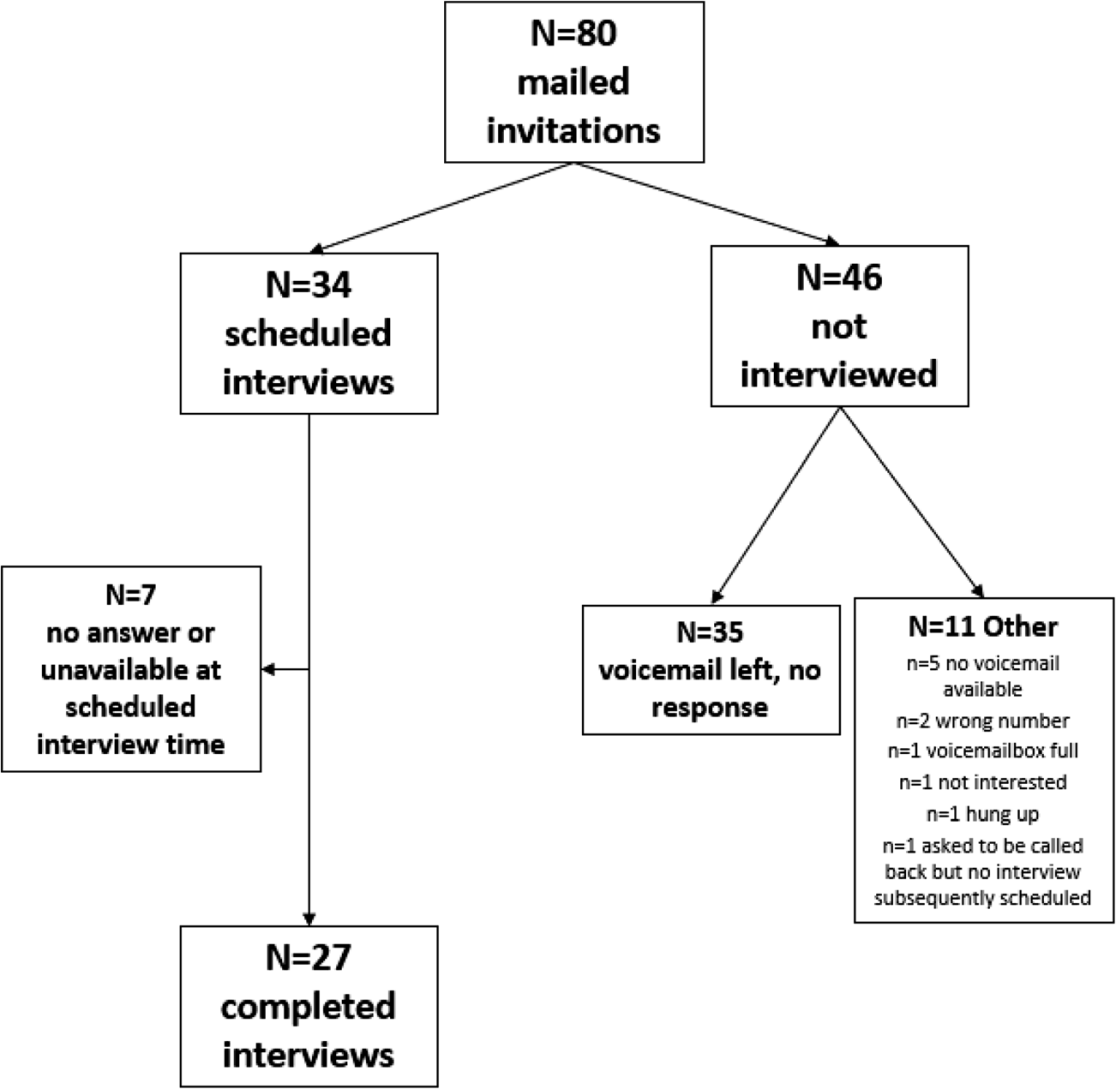
Participant Enrollment

We purposively sampled Veterans enrolled in the Center for Maternal and Infant Outcomes Research in Translation (COMFORT) cohort study.^11,23^ Veterans were eligible if they had delivered infants or were due by February 2020. Invitations to participate were mailed to 80 Veterans and included a phone number to opt-in; follow-up calls to participants who did not opt-in were made by study staff two weeks after invitations were sent. We scheduled 34 interviews and completed 27 (34% of all invited participants; Fig. 1). Interviews took place in December 2019. Those who participated in an interview received a $25 gift card for their time. This project was approved by the VA Central Institutional Review Board.

### Participant Characteristics

Sample characteristics were obtained from two interviews completed by participants as part of their enrollment in the COMFORT study: a prenatal interview (~16–20 weeks pregnant) and a postpartum interview (≤3 months after delivery). Characteristics reported here include age at prenatal interview; self-reported race; ethnicity; marital status; urban or rural residence; first pregnancy; receipt of VA maternity care coordination services; mental healthcare at the beginning of pregnancy; self-reported past diagnosis of depression, anxiety, or PTSD; history of military sexual trauma; recent intimate partner violence; and prenatal EPDS score. The EPDS is a 10-item depression symptom screener validated for use in a pregnant population, with scores of 10 or higher indicative of depression symptoms.^24,25^ Military sexual trauma (MST) was assessed by an adapted version of the VA’s universal MST screening questions.^26^ Intimate partner violence (IPV) in the 12 months prior to pregnancy was measured using the Extended Hurt Insult Threaten Scream Tool (E-HITS),^27^ where a score of ≥7 indicated IPV.^28^ For women who had delivered, we report birth outcomes including baby birthweight and delivery mode.

### Interview Process and Analysis

Interviews followed a semi-structured interview guide (Appendix A) and were conducted by one interviewer (AKD) until it was determined that theoretical saturation was reached.^29^ Interviews were roughly 30 minutes each, took place by phone, were digitally recorded to capture verbatim content, and professionally transcribed.

Two coders participated in the analysis (AKD, RK). We utilized framework analysis with an inductive approach to understand our data, interpret and code our transcripts, and develop themes.^30–32^ Data were managed using ATLAS.ti version 8.^33^ We anonymized participant names with pseudonyms and used anonymous participant numbers to correlate to their quantitative data. To triangulate our data, we examined mental health characteristics of Veterans who supported the USPSTF recommendations of mental health counseling at the beginning of pregnancy compared to those who were unsure or did not support these recommendations (quotes indicating support or not for the recommendations are found in Appendix B).

## RESULTS

### Sample Characteristics

Among our 27 participants, 10 were in their third trimester of pregnancy; 17 were postpartum. Participants represented nine VA medical centers, with 65% of our sample living in an urban area. On average, participants were 31 years old, 59% were white, and over half (56%) were married. Only 6 (22%) women were experiencing their first pregnancy. The majority (85%) of our participants did not show symptoms of depression on their prenatal EPDS. Fewer than half (44%) of the women reported seeing a mental health provider at the beginning of their pregnancy. Nineteen (70%) women self-reported being diagnosed with depression, anxiety, or PTSD in the past. More than 50% of our sample had experienced MST (Table 1). Our sample was similar to the overall COMFORT cohort with regard to age, race, ethnicity, marital status, history of MST, and past diagnoses of depression, anxiety, and/or PTSD (data not shown).

**Table 1:** Participant Characteristics (*N*=27)

	Total
Pregnancy status at interview, *N* (%)	
3rd trimester	10 (37.0)
Postpartum	17 (63.0)
Age (years), mean ± SD, range	31.3 ± 4.0 (24.6–36.6)
Race, *N* (%)	
White	16 (59.3)
Black	5 (18.5)
Other	8 (29.6)
Hispanic or Latino/Latina, *N* (%)	9 (33.3)
Married, *N* (%)	15 (55.6)
Rural/urban status, *N* (%)	
Rural	6 (20.7)
Urban	19 (65.5)
First pregnancy, *N* (%)	6 (22.2)
Received any VA maternity care coordination services, *N* (%)	25 (92.6)
Experience of military sexual trauma (harassment and/or rape), *N* (%)	17 (63.0)
Experience of IPV in the past year, *N* (%)	4 (14.8)
Delivery mode*, *N* (%)	
Vaginal	11 (68.8)
Caesarean	5 (31.2)
Baby birthweight* (pounds), *N* (%)	
<5.5lbs	1 (6.2)
≥5.5lbs to <9lbs	13 (81.3)
≥9lbs	2 (12.5)

Note: VA sites represented by participants included Boston, Dallas, Durham, Fargo, Los Angeles, Little Rock, Minneapolis, Tampa, and West Haven. Race is not mutually exclusive

*Data available for *n*=16 postpartum respondents. One participant did not complete a postpartum interview. Postpartum participants were between 4 days and 3.5 months postpartum

### Findings

Three main themes emerged from our interviews with perinatal Veterans. These themes are outlined in Table 2 and in detail below.

**Table 2:** Themes Identified by Veterans on USPSTF Mental Health Counseling Recommendations and Mental Healthcare During the Perinatal Period

Theme	Key findings
Veterans support USPSTF recommendations in the VA and consider mental healthcare to be very important during the perinatal period	- Immediate access to mental healthcare needed - Continuous relationship with one provider important - Importance of preventative care for challenges due to medication (e.g., antidepressants) discontinuation during pregnancy, family changes, physical changes, and postpartum expectations
Women Veterans would like better access to mental healthcare resources and peer support networks	- Dedicated resources for Veterans (e.g., phone helpline) - Veteran support groups desired as many civilian friends do not understand Veteran experiences - Support groups could be virtual
Pregnant and postpartum Veterans suggest that perinatal depression screening could be more extensive	- Comprehensive screening needed - Social desirability bias present during screenings

#### Theme 1

Veterans support USPSTF recommendations in the VA and consider mental healthcare to be very important during the perinatal period

Veterans in our sample were overwhelmingly in support of a mental health counseling referral at the beginning of pregnancy aligned with the USPSTF recommendations. As Lisa, 35 weeks pregnant, illustrated, “That would be awesome. I think (the VA) should really do that, like in the beginning. Don’t just push (pregnant Veterans) off to the side and wait for them to have to call.” Veterans specifically noted how mental healthcare may not be needed at all points during the perinatal period, but that having a relationship with a provider initiated at the beginning of pregnancy would provide access when needed, as “depression can kind of hit quickly” and “you don’t want to wait a month or two months” for an appointment. Kelly, who was 38 weeks pregnant, had similar thoughts: “It would be good to just touch base…not necessarily that you need to keep going, but just to know that it’s there.” Similarly, Sarah, who was 3 weeks postpartum, discussed how mental health counseling should be more widely available, but not necessarily mandatory: “Some people don’t need it and some people do. I do think that Veterans are more high-risk, especially female Veterans who have experienced combat and non-combat related trauma at a much higher rate than the general population. I think it’s good to have it be offered, at least.”

Of the five Veterans who said they would not want to be referred to counseling or were unsure what they would do if it was offered, reasons for not supporting the recommendations included not wanting an extra appointment when also receiving prenatal care, not wanting to be singled out because of their Veteran status, and having enough social support from friends and family. There were few differences in mental health characteristics between our participants who did and did not endorse the recommendations, including existing mental health counseling and diagnosis of past depression (Table 3).

**Table 3:** Mental Health Characteristics of Participants by Support for USPSTF Mental Health Counseling Recommendations

	Supportive *N*=22	Unsure/not supportive *N*=5
Mental healthcare at the beginning of pregnancy, *N* (%)		
Seeing a VA mental health provider	9 (40.9)	2 (40.0)
Seeing non-VA mental health provider	1 (4.6)	0 (0.0)
Not seeing a mental health provider	12 (54.5)	3 (60.0)
Planned to continue seeing a mental health provider during pregnancy, *N* (%)	9 (40.9)	2 (40.0)
Self-reported past diagnoses, *N* (%)		
Depression	14 (63.6)	3 (60.0)
Anxiety	12 (54.6)	2 (40.0)
PTSD	3 (40.9)	3 (60.0)
Any of the above	16 (72.7)	3 (60.0)
Prenatal EPDS score ≥10, *N* (%)	2 (9.1)	2 (40.0)

Note: Appendix B includes quotes from participants indicating support or not for the recommendations

Throughout the discussion of the USPSTF recommendations, it was evident that Veterans consider mental healthcare to be very important during the perinatal period. Seven Veterans shared their experiences of perinatal depression or anxiety and how that made them realize the importance of mental healthcare during the perinatal period. Elena, a first-time mom, said “it’s a delicate time, and it’s important to seek help, especially if you feel like you need it” and noted that she “didn’t realize how scary” postpartum depression was until she experienced it*.* Catherine, who was 38 weeks pregnant, elaborated: “I got really bad depression after my first son…I was really depressed. You know, people say if you want to kill yourself, ‘think about the people you’re leaving behind’. But that’s how depressed I was after my son, you don’t care. But I feel like if I had an appointment or something that could have helped me.”

Veterans also spoke about preventative care and how having earlier care could potentially ease any emerging symptoms. As Claire, who was 3 months postpartum, put it, “prevention is a really, really good idea. Even though I didn’t have that many problems, I still think I would have benefitted from (counseling). I think anyone would… Sometimes you’re thinking about things that you don’t want to tell everybody else, because you don’t want to worry them or make them think—‘Oh, gosh, she’s going to have postpartum (depression)’ or ‘she’s depressed’ or whatever. Sometimes you just want to talk.” Carolyn, who was 2 months postpartum, specifically noted how important mental healthcare could be for Veterans who had adjusted their medications due to pregnancy: “I was off of my medication, so having somebody to talk to once a week or every other week, you know, that could have helped me maybe process my thoughts about maybe something I was thinking about. Or, you know... if my anxiety was up at that time, just having that person to talk to would have been a great option.” Three Veterans noted specifically that they would only be interested in mental healthcare if it was accessible. This included having evening and weekend appointments available to make counseling more feasible for women with full-time jobs and/or other children at home.

Other Veterans discussed how physical changes during pregnancy along with family and life changes could affect mental wellbeing. Expectations of the postpartum period and having a newborn were also brought up, with Veterans noting how challenging the postpartum period can be and how important it is to have mental healthcare during this time. As Angelina, a first-time mom who was 7 weeks postpartum, said: “You know, nobody talks about postpartum expectations or what to watch out for or anything like that. I had a lot of things in place to help me through that. And I did a lot of learning beforehand, so I was better prepared. But I can’t imagine if I hadn’t been.”

#### Theme 2

Women Veterans would like better access to mental healthcare resources and peer support networks.

Veterans suggested a need for more resources dedicated to mental health and perinatal depression care. In particular, a helpline that Veterans could call to have an easy way to access a mental health provider when needed for issues specifically related to perinatal mental health was mentioned. Anne, who was 39 weeks pregnant, said that “it would be nice to have somebody to call or contact right away... ‘Hey, here’s a 24-hour help line, if something does happen’. That would be good to provide to people... (the National Suicide Prevention Hotline) doesn’t really focus on the issues involved with postpartum care.”

Others spoke about the need for the VA to be more transparent about available resources, specifically regarding what was available at their local VA facility as well as in the community. As Elena, who was 1 month postpartum, said, “I just think it’s important for first time moms to understand that these options are available to us, like the counseling and everything.” Virtual options may make care more accessible during pregnancy and the postpartum, especially as 20% of our sample lived in a rural area (Table 1). While these interviews took place prior to the advent of the COVID-19 pandemic when options for telehealth were more limited for Veterans, telehealth was discussed by our participants. Julia, who was 3 months postpartum, stressed how important it was to have consistency with providers regardless of the delivery mode for care when she said, “I’d say the key to that is it has to be the same person. That’s how you build trust. I think the main thing, if you’re doing it remotely, is if it’s the same person or not.”

Additionally, participants expressed a desire to talk with other Veterans also experiencing pregnancy, either with in-person or virtual support groups. Participants expressed that Veteran-specific support groups would be beneficial as many civilian friends do not necessarily understand military experiences and how that may affect perinatal mental health. As Elena put it, “I haven’t heard or been offered support groups. I think I would benefit a lot from that. I know a lot of other women would, too.” Other women noted feeling isolated as a Veteran and wanting other women Veterans to talk to. Jennifer, who was 3 months postpartum and a first-time mom said, “Being alone and being kind of in the position where you don’t have anybody to talk to… just being able to talk to other people who are experiencing the same thing would be nice. Group activities that would make you feel more normal and I’m not alone in this and other people are going through the same thing.” Naomi, who was 1 week postpartum, concurred: “I mean, there are so many options out there for soldiers with PTSD and bipolar or any kind of mental disability that they may have acquired while they were in service. There’s plenty of choices out there, plenty of groups out there, but it seems like the women Veterans who are now experiencing a whole other level of stress and pressure on the body, have no one.”

#### Theme 3

Pregnant and postpartum Veterans suggest that perinatal depression screening could be more extensive

A final theme that emerged was Veteran’s desires to have more comprehensive perinatal depression screening. When asked if they remembered receiving a depression screening during pregnancy, Veterans recalled only quick screenings, and several (*n*=4) specifically stated that the perinatal depression screenings they received could have been conducted in greater depth. Twelve participants recalled that they received at least one screening from their VA maternity care coordinator but noted that there were very few screening questions. As Anne, who was 39 weeks pregnant, put it, “I mean, they went down this checklist and asked me some questions about it.” Audrey, who was 1 month postpartum, said: “It wasn’t very good screening. They’re asking you—‘Do you feel tired?’ Uh, duh. Of course I feel tired, I have two kids. ‘Have you lost interest in doing things you love?’ Uh, yeah. I have two kids. There’s a difference between energy level and a mental issue. And of course you’re not going to admit—‘Oh, I have postpartum depression’. Because in your mind, you’re just finding it as normal, because… (having a newborn) is difficult.”

Three Veterans recommended that more comprehensive perinatal depression screening should be conducted by someone with mental health training. Rose, who was 38 weeks pregnant, shared: “(A mental health provider) would actually ask the right questions, instead of just vaguely asking if you’re depressed.” Veterans also noted that screenings may not have been accurate due to social desirability bias and feeling like they needed to be positive as new mothers to the providers they were speaking with. Catherine said: “You know, they’re saying things like—‘I bet you’re really happy and enjoying your baby.’ Like, yeah I am, but… I just remember she was all cheery and happy like—‘Oh, you should be so happy right now.’ And it’s like—Well, I’m not. So I felt bad.” Provider training on the administration of screening and in the compassionate navigation of diverse perinatal experiences may help destigmatize perinatal mental health conditions for Veterans in VA care.

## CONCLUSIONS

Since 1996, when the VA first authorized maternity care for women, few interventions have focused specifically on the mental health needs of pregnant Veterans.^34^ To begin to address this issue, our study presented pregnant and postpartum Veterans with a set of recommendations that would serve to substantially improve perinatal mental healthcare for Veterans. Our study participants advocated for more comprehensive perinatal depression screening and were widely accepting of an intervention within the VA based on USPSTF recommendations to prevent perinatal depression through a mental health counseling referral at the beginning of pregnancy.

Based on the recommendations as written, it is likely that a sizeable proportion of Veterans would be referred to counseling at the time of pregnancy confirmation using the USPSTF definition of “increased risk of perinatal depression” criteria.^9^ In this sample, over 70% of women had experienced either major depression, PTSD, or anxiety in the past and more than half had experienced MST. Additionally, there was strong support for mental health counseling during pregnancy despite only 15% of women showing symptoms on a prenatal EPDS and fewer than half already being engaged with mental healthcare at the time of pregnancy confirmation.

Further research on the resource implications for an intervention based on the USPSTF recommendations and tailored for VA care is warranted. An initial place to intervene may be in perinatal depression screening, where screeners validated for use in pregnant and postpartum populations (e.g., the EPDS) would be utilized instead of the Patient Health Questionnaire,^35,36^ which is used most frequently within the VA.^22^ However, understanding the availability of staff for more rigorous screenings and for mental health counseling is needed. Maternity care coordinators commonly serve as a bridge between pregnant and postpartum Veterans and their VA primary care provider, but often serve in this role secondary to their primary position within the VA, and more time-intensive care may prove burdensome. Similarly, resource allocation for mental health counseling services would need to be determined, and the economic impact of any new policy encouraging preventative perinatal depression care within the VA would need to be assessed. However, past research examining the economic burden of perinatal depression in general suggests that treating the disease is much costlier than prevention.^37^

The USPSTF guidelines recommend two specific evidence-based prevention interventions that could be potentially implemented into the VA, including the ROSE (Reach Out, Stay Strong, Essentials for mothers of newborns)^38,39^ and Mothers and Babies programs,^40^ both of which involve group educational sessions and can be taught by nonmental health specialists, increasing the available capacity of provider resources.^41^ Other approaches included in the USPSTF systematic review that informed their guidelines utilized individual and group counseling that were not part of a larger intervention.^10^ Within the VA, there is substantial evidence on the effectiveness of collaborative care for depression treatment through Primary Care-Mental Health Integration (PC-MHI) models.^42–46^ PC-MHI models have been associated with greater treatment initiation^45,47^ and comparable quality of depression care in the primary care environment, easing the demand on specialty mental health services.^48^ Existing PC-MHI structures could potentially be customized to meet the needs of a perinatal population and may provide an avenue for depression care and counseling throughout the perinatal period.

Our study is not without limitations. While the characteristics of our sample are comparable to those of women Veterans of similar ages in terms of race, urban/rural status, and mental health,^49^ we acknowledge that the results of this qualitative inquiry are limited by the purposive sampling done within a larger cohort study at selected VA medical centers and are therefore not generalizable to all pregnant and postpartum Veterans. Though we could not assess the impact of pregnancy outcomes in this analysis, an established relationship with a mental health counselor would also likely be helpful for instances of severe maternal morbidity, including pregnancy complications such as preterm labor or preeclampsia, and other post-delivery stressors that may cause mental distress.^50^ Other factors, such as maternity care coordinator involvement or other site-specific resources (e.g., mental healthcare appointment availability, provider training in perinatal mental health), may have influenced our findings. Nonetheless, this study is an important foundation to understand the appropriateness of a counseling intervention and to build an evidence base for future implementation work.

Engaging Veterans with mental healthcare upon pregnancy confirmation may initiate a relationship between the Veteran and VA mental health services, should care be needed at some point during the perinatal period. Universal referral would also mitigate the challenge of identifying new-onset perinatal depression and reduce stigma associated with seeking treatment for symptoms during pregnancy and the postpartum period. Understanding the real-world feasibility and prevailing barriers to comprehensive perinatal depression care is needed to inform implementation of the USPSTF recommendations or a similar intervention tailored for VA care. Research should now focus on assessing organizational readiness and determining which preventative interventions would be most feasible within VA care.

## Supplementary Information

ESM 1 (DOCX 16 kb)

ESM 2 (DOCX 18 kb)
