# Supplementary material for: Exploring the Acceptability of Expanded Perinatal Depression Care Practices Among Women Veterans
Source: J Gen Intern Med. 2022 Aug 30;37(Suppl 3):762–9. doi: 10.1007/s11606-022-07573-7 (PMC9427169; doi:10.1007/s11606-022-07573-7)
Supplement: Supplementary file 1 — (DOCX 16 kb) [file 11606_2022_7573_MOESM1_ESM.docx]

**Appendix A. Semi-Structured Interview Guide**

1. To start, I’d like to congratulate you on your recent (pregnancy/baby)! How has everything been going?
2. I’m curious in learning more about mental health care during your pregnancy and in the postpartum.
   1. Prompt: How often were you screened for depression symptoms during pregnancy? Do you recall being screened for depression during pregnancy? How often? By who?
   2. Prompt: Did you have any discussions with your providers (mental health or other) about any mental health medications you may have been on before you became pregnant? If you discontinued any medications, was this a decision that was made by you, your provider, or through mutual decision-making process? Do you feel like you were given enough information on medication and/or medication alternatives for mental health during pregnancy?
   3. Prompt: During conversations with your provider/maternity care coordinator regarding mental health care, were you offered or made aware of treatment including counseling or other services that were available to you?
   4. Prompt: Was/is your obstetrician aware that you are a veteran?
3. Thinking about mental health care treatment specifically, there is a chain of events that must occur for someone to receive adequate care. This starts with screening, continues to treatment (if needed), and symptom monitoring, where someone in care would meet with a provider regularly to make sure their treatment is working for them.
   1. Prompt: During any mental health care you’ve received at the VA, did your care progress from screening to monitoring? If yes, could you describe this care? If no, where did you feel like more attention was needed?
4. How has your transition from OB care back to VA care been? Do you feel like you have been offered support with this transition? Does the VA know about complications you experienced during pregnancy/delivery?
5. In 2019, a group of doctors known as the United States Preventive Services Task Force recommended that women who are at increased risk of depression during their pregnancies and postpartum be referred to counseling interventions to prevent episodes of depression. Veterans are often considered high-risk due to their previous experiences with combat and other traumas.
   1. Prompt: Did you have an existing relationship with a VA mental health provider prior to your pregnancy? If so, did you have any contact with this provider during your pregnancy or in the postpartum? If not, how would you have felt about being referred to a mental health provider at the beginning of your pregnancy? Would you have been open to being referred to a mental health provider at the beginning of your pregnancy?
   2. Prompt: Did you continue to receive any care at the VA or with a VA provider during your pregnancy? What did that care look like?
   3. Prompt: If you were not engaged in VA mental health care, would you have been open to speaking with a therapist? If this was offered at the beginning of your pregnancy, would it have been helpful to receive counseling in-person, by telephone, or group therapy?
