# Supplementary material for: Exploring the Acceptability of Expanded Perinatal Depression Care Practices Among Women Veterans
Source: J Gen Intern Med. 2022 Aug 30;37(Suppl 3):762–9. doi: 10.1007/s11606-022-07573-7 (PMC9427169; doi:10.1007/s11606-022-07573-7)
Supplement: Supplementary file 2 — (DOCX 18 kb) [file 11606_2022_7573_MOESM2_ESM.docx]

Appendix B.

| **Pseudonym** | **Pregnant or Postpartum** | **First Baby** | **Quote Regarding USPSTF Recommendations (Mental Health Counseling) at the Beginning of Pregnancy*** | **Supportive of Recommendations** |
| --- | --- | --- | --- | --- |
| Dora | Postpartum | No | *Yes, because (Veterans) would get the help they need and they (wouldn’t) have to worry about if they need it or not, because they already had everything set up, so all they need to do is just make a phone call, make an appointment, and go to the clinic.* | Yes |
| Angelina | Postpartum | Yes | *It might be really helpful for (Veterans) to have some kind of therapy throughout their pregnancy to help with the hormonal changes.* | Yes |
| Amanda | Postpartum | No | *I’d definitely be okay with it.* | Yes |
| Theresa | Pregnant | No | *I don’t know. Probably. But I mean, you know, I was fine.* | No/unsure |
| Anne | Pregnant | No | *Well, I could see how some Veterans—it might be useful. But for me it’s like, I don’t want to be forced to go see a counselor when I don’t have the real need for it, you know? If that makes sense. It’s just another appointment, another person to talk to. Then I go through an appointment and then have to sit there and tell them—I’m fine. We’ll be fine.* | No/unsure |
| Jennifer | Postpartum | Yes | *That would be great. Because that seems to be the hardest thing to do, trying to reach someone, schedule an appointment and then a lot of times the appointments are like two or three months from the time you call.* | Yes |
| Jessica | Postpartum | No | *(It would be) better for somebody like me. To try and avoid it getting as bad as it can get before you reach out for help.* | Yes |
| Sarah | Postpartum | Yes | *Having it be offered would be helpful. But I mean, obviously, some people don’t need it and some people do…I think it’s good to have it be offered, at least.* | Yes |
| Catherine | Pregnant | No | *Definitely. I think counseling should be like—I don’t want to say forced—but I feel like, you should (go).* | Yes |
| Julia | Postpartum | No | *I wouldn’t mind, if I needed it. But it would really annoy me if I had to like go and get—you know, do an extra step and get screened by a psychologist, just because.* | No/unsure |
| Liz | Pregnant | No | *I wouldn’t like it. If it’s based solely on just because of my Veteran status, it seems like I’d just be singled out because of that. And, I don’t know, that just kind of puts a bad taste in your mouth and a negative connotation on a Veteran.* | No/unsure |
| Kelly | Pregnant | Yes | *I think that’s great, because I think a lot of people—regardless of whether they’re Veteran or not—are not comfortable, necessarily, with talking about mental health. So, if you just put it out there and they have that option where it’s already set up and they can open up about it, that’s always a good thing.* | Yes |
| Heather | Postpartum | No | *I think having that available as an option for (Veterans) is great.* | Yes |
| Claire | Postpartum | No | *Prevention is a really, really good idea. And even though I didn’t have that many problems, I still think I would have benefited from it. I think anyone would.* | Yes |
| Elena | Postpartum | Yes | *I think it’s necessary. I think any friend that I’ve had that’s gone through pregnancy or has already had their baby, they all—I feel like they need some extra support.* | Yes |
| Alyssa | Pregnant | No | *I think that’s a great idea. Not only because of like the military aspect, but like everyday stressors, you know, work, and having other children and relationship issues… I think it would definitely help to make a smoother transition throughout the rest of the pregnancy.* | Yes |
| Sadie | Postpartum | No | *I think it’s really good.* | Yes |
| Naomi | Postpartum | No | *Yeah, I think that that’s definitely something that would help a lot of women Veterans.* | Yes |
| Audrey | Postpartum | No | *I do think it’s a good idea that the VA would offer something like that.* | Yes |
| Jean | Postpartum | Yes | *Yeah, I think it (would be) very helpful.* | Yes |
| Rose | Pregnant | No | *I don’t think I would have taken it at the beginning, because I haven’t had No—I don’t feel depressed during my pregnancies. Maybe after, but not during. (I would) consider during.* | No/unsure |
| Lisa | Pregnant | No | *I think that would be awesome. I think they should really do that, like in the beginning. Don’t just push them off to the side and wait for them to have to call. Because the beginning is hard.* | Yes |
| Ruth | Pregnant | No | *I think it’s a good idea if they make the counseling accessible.* | Yes |
| Carolyn | Postpartum | No | *I think it’s important. I think it’s important to offer that to (Veterans) from the beginning of pregnancy. I think it would be beneficial to Veterans.* | Yes |
| Mindy | Postpartum | No | *I see positive things from that. That would probably help a lot. Because it did help me a lot. I mean, I didn’t get it at the start, but the fact that I had—you know, mental health at the middle, helped out a lot. And it probably would have helped me out at the start a lot with the anxiety that I was having and you know, the mental issues that I was having at the beginning, the worries and stuff. So I think it would probably be very helpful.* | Yes |
| Nicole | Pregnant | Yes | *It would be good, but from what I understand, there’s not as many women in the VA. I think it would be better to do like a teleconference or something similar to that, as an emotional support group or something like that.* | Yes |
| Madeline | Postpartum | No | *This is a good proposition to have. (Because) people don’t really feel comfortable talking about it, going to see someone.* | Yes |

**The USPSTF recommendations were described and Veterans were asked: “How would you have felt about being referred to a mental health provider at the beginning of your pregnancy?” or “**Would you have been open to being referred to a mental health provider at the beginning of your pregnancy?”*
